# Alumni Association

**Published:** 1879-05

**Authors:** 


### ALUMNI ASSOCIATION.

The alumni of the Dental College of the University of Michigan, convened at Ann Arbor, March 26, and after due consideration, proceeded to the formation of a permanent organization, to be known by the name above indicated.

The following officers were chosen for the ensuing year, viz:

President, Dr. D. C. Hawxhurst, of Battle Creek, Mich.

Vice-President, Dr. D. S. Shattuck, of Detroit.

Secretary, Dr. B. F. Miller, of Flint.

Treasurer, Dr. H. F. Harvey, of Cleveland, O.

The Executive Committe consists of the above named officers, with the addition of Dr. W. H. Jackson, of Ann Arbor.

This committee was instructed to prepare a constitution and by-laws and report the same at the next meeting of the association, on the evening of the last Wednesday of March, 1880.

This it is to be hoped is the beginning of that which will be permanent and profitable.

